# Avian leukosis virus subgroup J induces VEGF expression *via* NF-κB/PI3K-dependent IL-6 production

**DOI:** 10.18632/oncotarget.13282

**Published:** 2016-11-10

**Authors:** Yanni Gao, Yao Zhang, Yongxiu Yao, Xiaolu Guan, Yongzhen Liu, Xiaole Qi, Yongqiang Wang, Changjun Liu, Yanping Zhang, Honglei Gao, Venugopal Nair, Xiaomei Wang, Yulong Gao

**Affiliations:** ^1^ Division of Avian Infectious Diseases, State Key Laboratory of Veterinary Biotechnology, Harbin Veterinary Research Institute, Chinese Academy of Agricultural Sciences, Harbin, China; ^2^ Avian Viral Diseases programme & UK-China Centre of Excellence on Avian Disease Research, The Pirbright Institute, Ash Road, Pirbright, Surrey, GU24 0NF, UK

**Keywords:** ALV-J, interleukin 6, tumorigenesis, VEGF-A, VEGFR-2, Immunology and Microbiology Section, Immune response, Immunity

## Abstract

Avian leukosis virus subgroup J (ALV-J) is an oncogenic virus causing hemangiomas and myeloid tumors in chickens. Interleukin-6 (IL-6) is a multifunctional pro-inflammatory interleukin involved in many types of cancer. We previously demonstrated that IL-6 expression was induced following ALV-J infection in chickens. The aim of this study is to characterize the mechanism by which ALV-J induces IL-6 expression, and the role of IL-6 in tumor development. Our results demonstrate that ALV-J infection increases IL-6 expression in chicken splenocytes, peripheral blood lymphocytes, and vascular endothelial cells. IL-6 production is induced by the ALV-J envelope protein gp85 and capsid protein p27 *via* PI3K- and NF-κB-mediated signaling. IL-6 in turn induced expression of vascular endothelial growth factor (VEGF)-A and its receptor, VEGFR-2, in vascular endothelial cells and embryonic vascular tissues. Suppression of IL-6 using siRNA inhibited the ALV-J induced VEGF-A and VEGFR-2 expression in vascular endothelial cells, indicating that the ALV-J-induced VEGF-A/VEGFR-2 expression is mediated by IL-6. As VEGF-A and VEGFR-2 are important factors in oncogenesis, our findings suggest that ALV-J hijacks IL-6 to promote tumorigenesis, and indicate that IL-6 could potentially serve as a therapeutic target in ALV-J infections.

## INTRODUCTION

Avian leukosis virus (ALV) is a highly oncogenic alpha-retrovirus of Retroviridae family, causing avian leukosis (AL) in chickens. ALVs can be classified as endogenous (ALV-E) and exogenous (A, B, C, D, and J) based on their mode of transmission, host range, viral envelope interference, and cross-neutralization patterns [1, 2]. ALV-J was first isolated from meat-type chickens in 1988 [2], and has primarily been associated with myeloid leukosis (ML) in broiler breeders [3, 4]. In recent years, however, various tumors including hemangiomas induced by ALV-J have emerged among parent and commercial layer flocks [5, 6], leading to enormous economic losses, and indicating an evolution of ALV-J oncogenicity.

Tumorigenesis is a complex process caused by a variety of factors. Some viruses contain oncogenes in their genomes; therefore induce tumors *via* the multiple functions of these oncogenes. However, ALV-J does not carry a viral oncogene. Most studies regarding the ALV-J oncogenicity have focused on the insertional mechanisms of ALV-J, which activates or inactivates the tumor-associated genes of the host [7-11]. However, as ALV integrates in a largely random fashion with only a slight preference for active transcriptional units [12, 13], there must be some other mechanisms for ALV tumorigenicity.

It has been reported that VEGF-A and its receptor, VEGFR-2, are involved in ALV-J tumorigenesis [14]. VEGF is the most important proangiogenic agent that activates receptors on vascular endothelial cells (VECs) and promotes blood vessel regeneration. VEGF and VEGFR have been associated with the pathogenesis of leukemia. The VEGF/VEGFR-dependent pathways regulate angiogenesis, vasculogenesis, and recruitment of endothelial progenitor cells, and have been associated with progression and metastasis of solid tumors [15-17]. Furthermore, VEGF/VEGFR interactions may stimulate proliferation, migration, and survival of leukemia and lymphoma cells *via* autocrine and paracrine loops [18]. Notably, a previous study has indicated that acute leukemia cells secret large amounts of VEGF into the serum and that malignant hematopoietic cells express VEGF and VEGFRs [19]. We have previously shown that ALV-J infection induces expression of VEGF-A and VEGFR-2. A newly isolated ALV-J strain, with a stronger replication and oncogenesis capability, induced higher expression of VEGF/VEGFR in vascular cells and tissues than other ALV-J strains [14].

The expression of VEGF/VEGFR is associated with interleukin 6 (IL-6) signaling pathways in many cancers, such as breast and intestinal cancers [20, 21]. IL-6 is a multifunctional cytokine with central roles in immune and inflammatory reactions, as well as in cancer development [20-24]. IL-6 plays an important role in host immune system, wherein it has been considered to facilitate elimination of pathogens during virus-host interactions. However, through evolution, viruses have developed a number of strategies to avoid such an outcome and successfully establish chronic infections through hijacking the host immune system [25-27].

Our previous study has demonstrated that ALV-J infection promotes IL-6 expression *in vivo* in chickens [28]. Here, we tested the role of IL-6 in ALV-J-induced VEGF/VEGFR expression, and examined the underlying mechanisms.

## RESULTS

### ALV-J promotes IL-6 production in splenocytes, lymphocytes, and VECs

**Figure 1:**
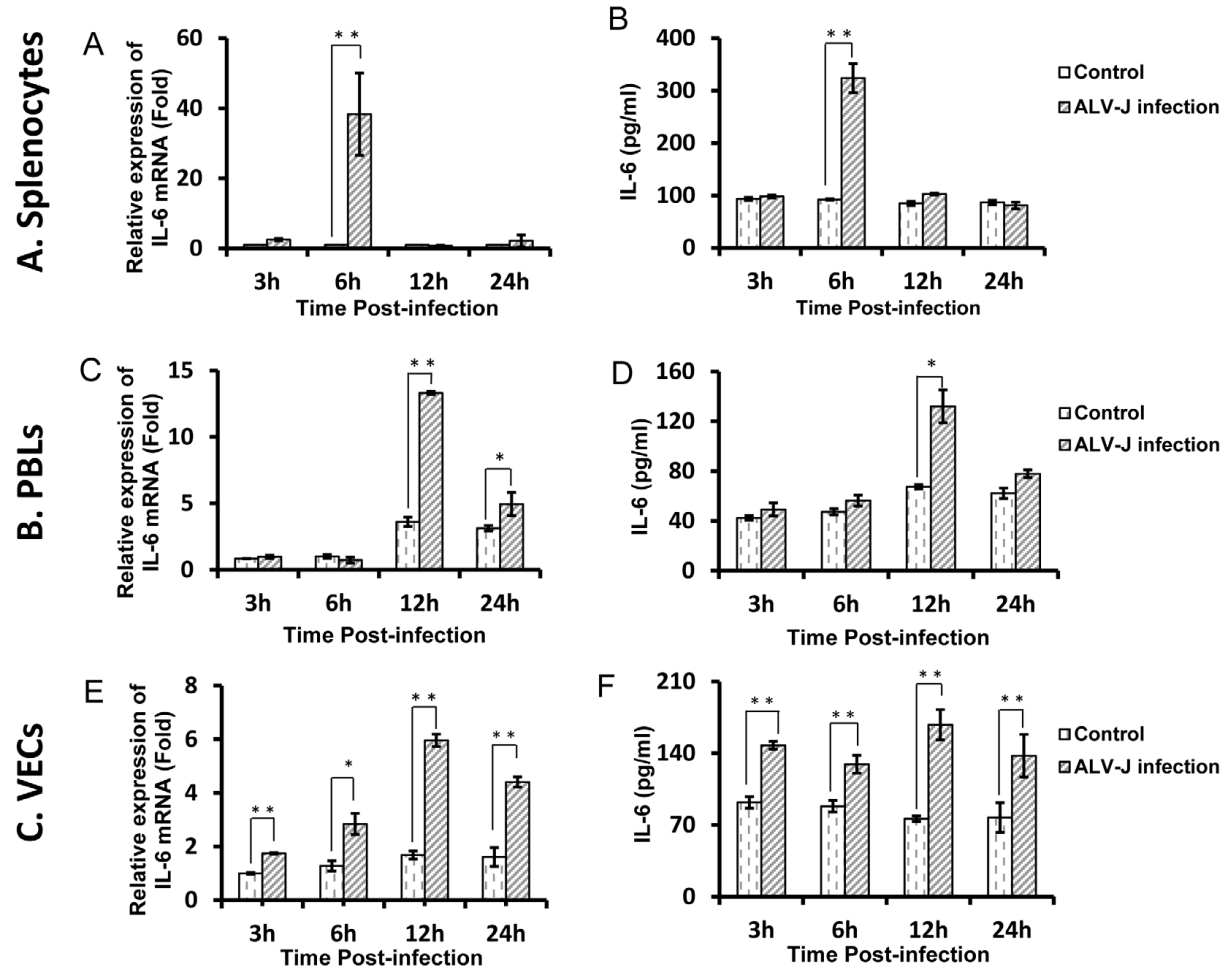
ALV-J promotes IL-6 expression *in vitro* **A.** Splenocytes, **B.** PBLs, or **C.** VECs were infected with ALV-J for 3, 6, 12, and 24 h and IL-6 mRNA and protein levels were determined using real-time RT-PCR (1) or ELISA (2).

We have previously shown that ALV-J promotes IL-6 expression *in vivo* [28]; in this study, we have investigated whether ALV-J induces IL-6 production *in vitro*. Chicken splenocytes, peripheral blood lymphocytes (PBLs), and VECs were infected with ALV-J strain HLJ09SH02. Enzyme linked immunosorbent assay (ELISA) was performed to confirm the successful ALV-J infection in these three cell types (data not shown). Infection with ALV-J strain HLJ09SH02 significantly induced IL-6 mRNA and protein levels in all three cell types (Figure 1). For splenocytes, the expression of IL-6 peaked at 6 h post-infection, with almost a 40-fold higher level compared to controls (*p* < 0.01). However, at 3, 12, and 24 h post-infection, the infected group showed no significant difference in IL-6 expression compared to control group (Figure 1A and 1B). For PBLs at 3 and 6 h post-infection, the IL-6 levels were similar between infected and control groups. At 12 h post-infection, IL-6 mRNA expression in the infected group was approximately 4-fold higher than in the control group (*p* < 0.01), with a similar trend exhibited for protein expression (*p* < 0.01) (Figure 1C and 1D). In VECs, the IL-6 expression differences appeared from 3 h post-infection and were maintained over the following 22 h. The expression of IL-6 mRNA in infected VECs peaked at 12 h post-infection, at a level of almost 3.5-fold higher than in the control cells (*p* < 0.01). ELISA results showed that IL-6 protein expression exhibited a similar trend (Figure 1E and 1F).

### ALV-J gp85 and p27 proteins promote IL-6 expression

**Figure 2:**
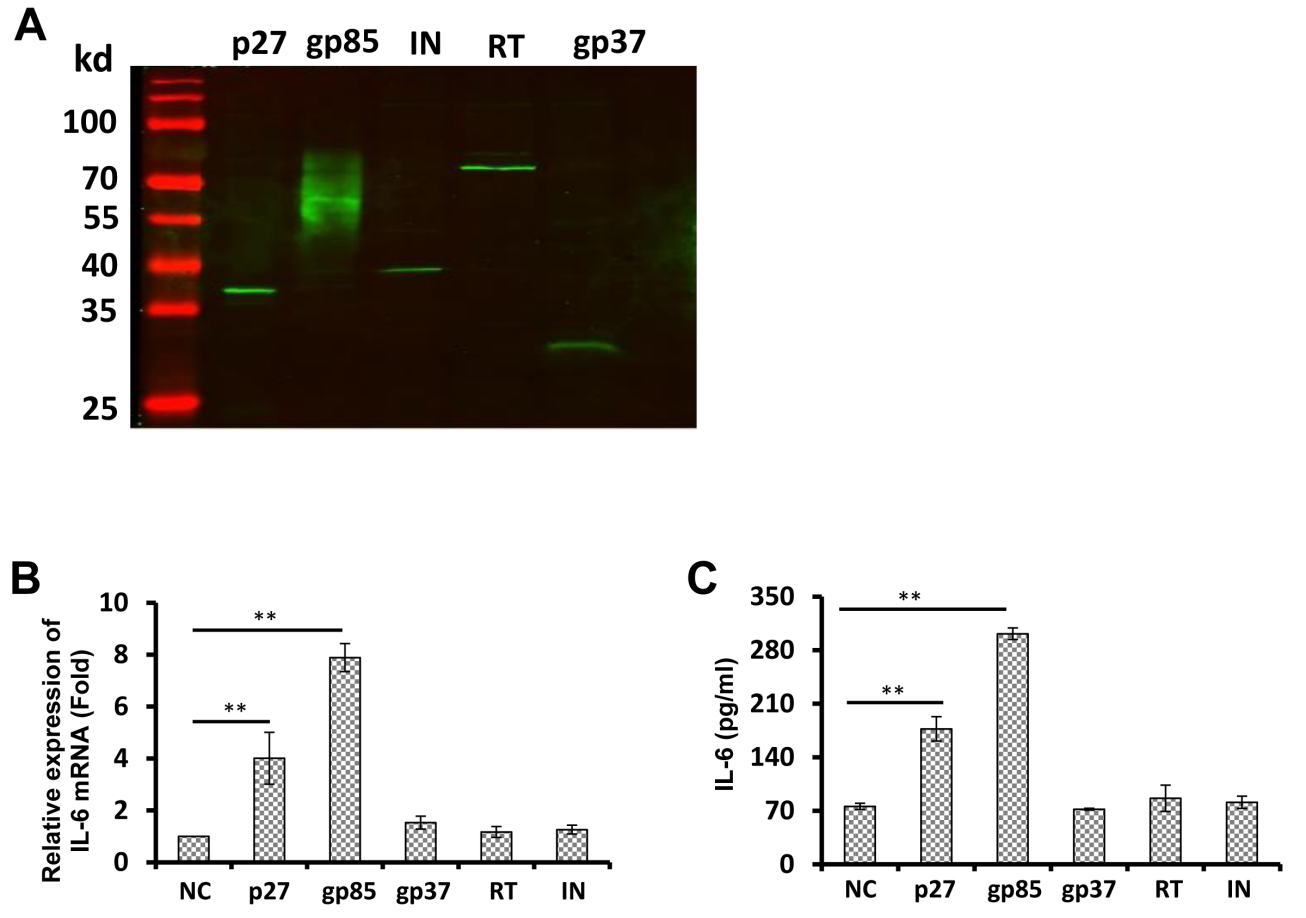
The ALV-J capsid protein p27 promotes IL-6 expression in a dose-dependent manner in splenocytes **A.** The expression of p27, gp85, integrase, reverse transcriptase, and gp37 were confirmed by western blot. Splenocytes were transfected with the pCAGGS vector or ALV-J p27, gp85, gp37, reverse transcriptase, or integrase expression vectors for 48 h. Then, *IL6* mRNA levels were determined using real-time RT-PCR **B.** and IL-6 protein levels were determined using ELISA **C.**

To examine whether the ALV-J-induced IL-6 expression was caused by ALV-J proteins, we analyzed the effect of p27, gp85, integrase, reverse transcriptase, and gp37 on IL-6 levels in splenocytes. Expression vectors of p27, gp85, integrase, reverse transcriptase, and gp37 were constructed, and the protein expression was confirmed by western blotting (Figure 2A). Splenocytes were then transfected with each respective vector. Cells and supernatants were harvested 48 h post-transfection for IL-6 mRNA and protein measurement by real-time reverse transcription-PCR (RT-PCR) and ELISA, respectively. The results showed that only gp85 and p27 increased the production of IL-6, with gp85 playing a more significant role. As shown in Figures 2B and 2C, ALV-J gp85 and p27 proteins increased IL-6 mRNA levels by approximately 8-fold and 4-fold, respectively, (*p* < 0.01). At protein levels, the increase was smaller, but still significant (*p* < 0.01). None of the other tested ALV-J proteins were able to increase IL-6 gene expression. These results indicate that ALV-J gp85 and p27 proteins promote the IL-6 expression.

### NF-κB and PI3K mediate ALV-J-induced chicken IL-6 expression

**Figure 3:**
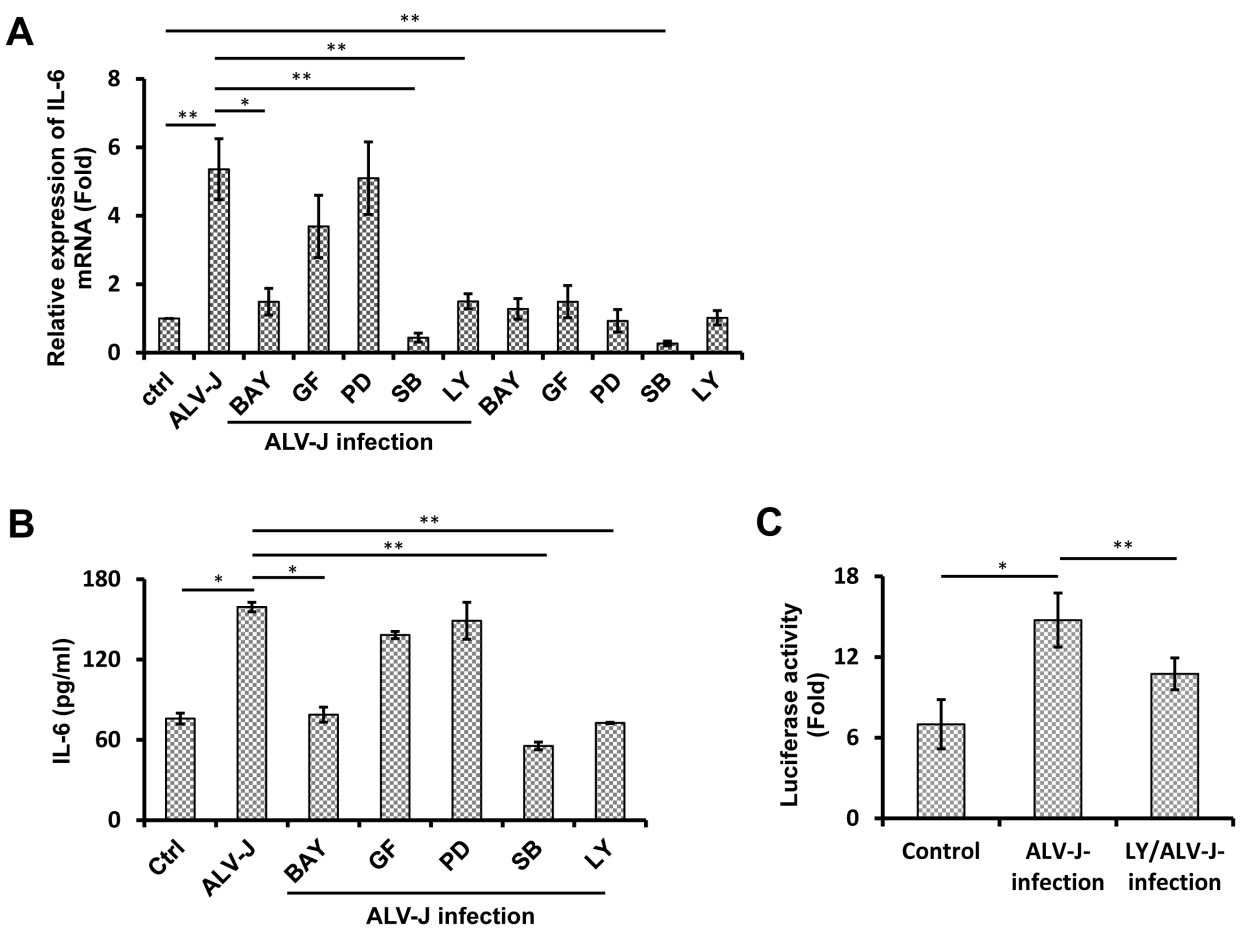
Effect of signal transduction inhibitors on IL-6 expression induced by ALV-J infection **A.**, **B.** Splenocytes were pretreated with inhibitors of NF-κB (BAY11-7082 [BAY]), PKC (GF-109203 [GF]), MEK (PD98059 [PD]), MAPK (SB202190 [SB]), PI3K (LY294002 [LY]), or DMSO control for 1 h and then inoculated with or without ALV-J (MOI = 0.1). After 6 h, total RNA was extracted for detecting *IL6* mRNA by real-time RT-PCR (A) and the cell culture supernatants were harvested to test IL-6 protein by ELISA **B. C.** Splenocytes were pretreated with LY or DMSO control for 1 h and then the pretreated splenocytes were transfected with plasmids to test NF-κB activity and infected with ALV-J (MOI = 0.1) or DMEM. After another 6 h, cells were harvested for luciferase analysis.

To dissect the signaling pathways involved in ALV-J-induced IL-6 production, splenocytes were pretreated for 1 h with inhibitors of key signaling pathways, including NF-κB, MEK, p38 MAPK, PI3K, or PKC prior to ALV-J infection. At 48 h post-infection, IL-6 expression was analyzed by real-time RT-PCR and ELISA. As shown in Figure 3A and 3B, NF-κB inhibitor BAY11-7082, PI3K inhibitor LY294002, and p38 MAPK inhibitor SB203580 significantly inhibited the IL-6 expression after ALV-J infection at both mRNA and protein levels, while inhibition of MEK (PD98059) and PKC (GF-109203X) exhibited no significant effect on the IL-6 production. However, a decrease in IL-6 production was also observed in cells treated with p38 MAPK inhibitor SB203580 without the ALV-J infection. These results suggested that p38 pathway was involved in the constitutive IL-6 expression in splenocytes, but NF-κB and PI3K pathways were specifically involved in the ALV-J-induced IL-6 expression.

To further verify these results and explore the relationship between NF-κB and PI3K pathways, a Dual-glo Luciferase Assay was utilized to test the activity of NF-κB with and without the activation of PI3K pathway. As illustrated in Figure 3C, although ALV-J was able to activate NF-κB on its own, NF-κB activity was decreased when activation of PI3K pathway was inhibited. This result suggested that activation of the PI3K signaling pathway was important for the ALV-J-induced NF-κB activation.

### IL-6 increases VEGF-A and VEGFR-2 expression *via* signal transducer and activator of transcription 3 (STAT3) signaling pathway

**Figure 4:**
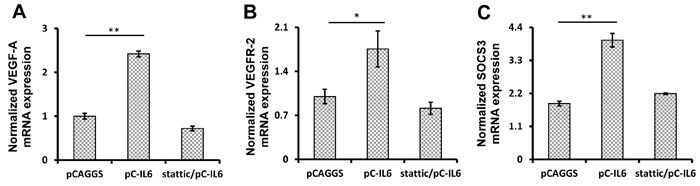
IL-6 promotes VEGF-A and VEGFR-2 expression *via* STAT3 signaling pathway in VECs *in vitro.* VECs were pretreated with an inhibitor of STAT3 (stattic) or DMSO control for 1 h. Then, the pretreated VECs were transfected with the pCAGGS vector or IL-6 expression vector for 48 h. Total RNAs were extracted and VEGF-A **A.**, VEGFR-2 **B.**, or SOCS3 **C.** mRNA expression was detected by real-time RT-PCR.

Previous studies have demonstrated that IL-6 serves as a link between inflammation and tumorigenesis, since it induces the expression of VEGF-A/VEGFR-2 [29]. As ALV-J is an important oncogenic virus, it was of interest to determine whether the ALV-J-induced IL-6 induces VEGF-A/VEGFR-2 expression in chickens. IL-6 gene expression vector was transfected into VECs to determine the effect of IL-6 on VEGF-A and VEGFR-2 expression. After 48 h post-transfection, VEGF-A expression was over 2-fold elevated compared to control cells transfected with empty pCAGGS vector (*p* < 0.01), and VEGFR-2 expression was nearly 1-fold elevated compared to control cells (*p* < 0.05) (Figure 4A and 4B). These results thus indicate that IL-6 induces VEGF-A and VEGFR-2 expression in VECs *in vitro*.

**Figure 5:**
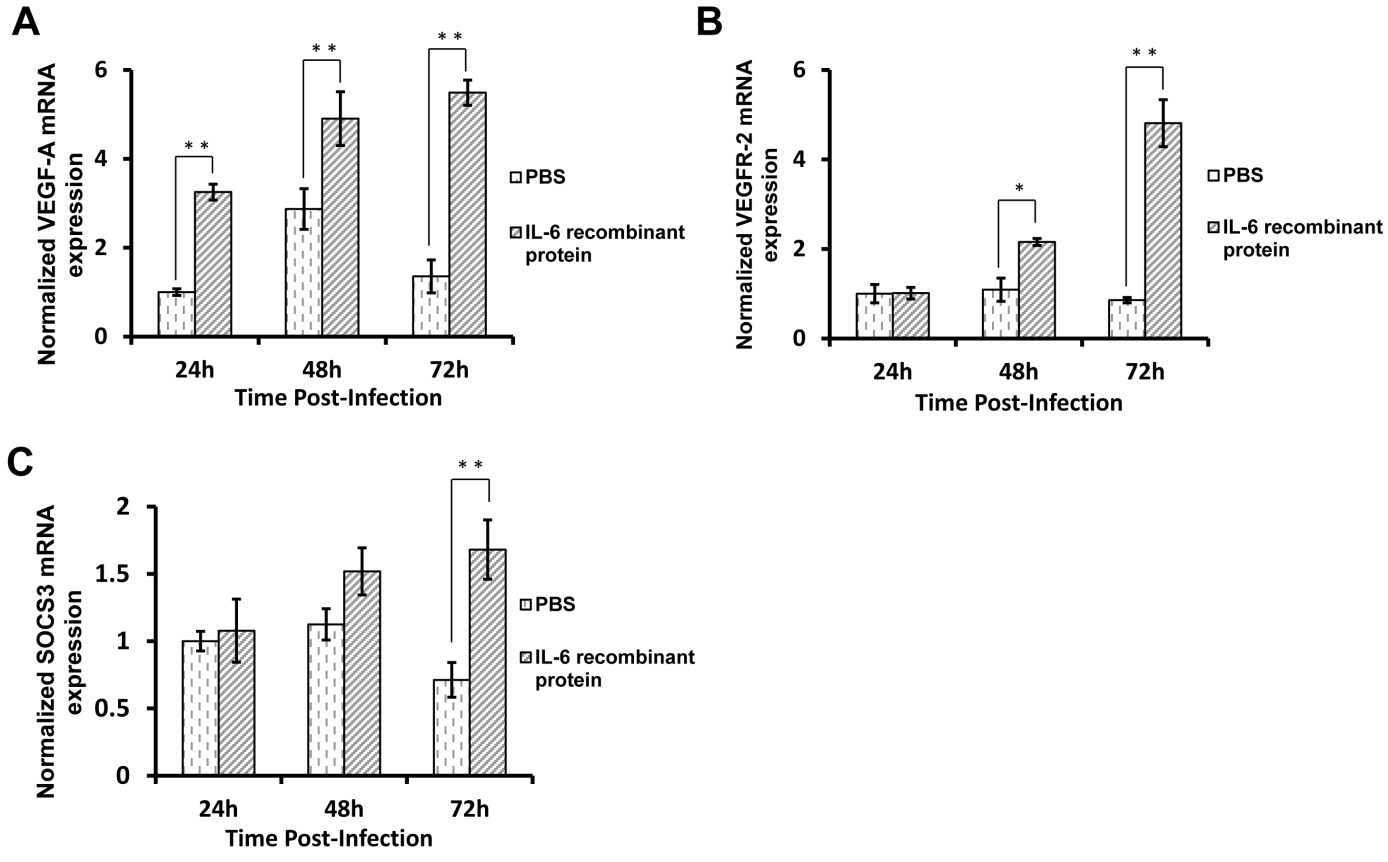
IL-6 promotes VEGF-A and VEGFR-2 expression by activating STAT3 in embryos *in vivo* **A.** Embryos were intravenously inoculated with IL-6 recombinant protein or PBS control. After 24, 48, or 72 h post-inoculation, the vascular tissues of the inoculated embryos were collected and used to extract total RNAs. Then, the levels of VEGF-A **A.**, VEGFR-2 **B.**, and SOCS3 **C.** mRNA were determined by real-time RT-PCR.

To validate these results *in vivo*, embryos were used to test the effect of IL-6 on VEGF-A/VEGFR-2 production. Recombinant IL-6 protein was inoculated into 7-day-old embryos intravenously, and vascular tissues were collected 24, 48, and 72 h post-inoculation. As shown in Figure 5A, VEGF-A expression in the inoculated embryos was significantly increased compared to control embryos 24 h post-inoculation (*p* < 0.01), and this difference became even more obvious over the following 48 and 72 h post-inoculation (*p* < 0.01). For VEGFR-2, at 24 h post-inoculation, VEGFR-2 expression showed no difference between inoculated and control embryos. At 48 h post-inoculation, VEGFR-2 expression in the inoculated embryos increased nearly 2-fold compared to control embryos (*p* < 0.05), with the difference reaching almost 5-fold at 72 h post-inoculation (Figure 5B). These results thus demonstrate that IL-6 increases VEGF-A and VEGFR-2 expression in embryos *in vivo.*

Previous studies have indicated that STAT3 represents a downstream signaling pathway of IL-6 [29-31]. In order to investigate the signaling pathways involved in the VEGF-A and VEGFR-2 regulation by IL-6, STAT3 inhibitor stattic was used to pretreat VECs prior to IL-6 transfection. The results showed that upon stattic pretreatment, VEGF-A and VEGFR-2 expression in IL-6-transfected VECs was reduced compared to cells transfected with control vectors (Figure 4A and 4B), indicating that STAT3 was involved in the IL-6-induced VEGF-A and VEGFR-2 expression. Suppressor of cytokine signaling 3 (SOCS3) is considered as an indicator of STAT3 activation as it is often over-expressed during STAT3 activation [32-34]. Therefore, to further verify the activation of STAT3, we analyzed the expression of SOCS3 in both VECs and vascular tissues of embryos. Real-time RT-PCR results indicated that upon IL-6 treatment, STAT3 signaling was activated both *in vitro* and *in vivo* (Figure 4C and 5C). These results suggest that STAT3 signaling is involved in the IL-6-induced VEGF-A and VEGFR-2 expression.

### ALV-J induces VEGF-A and VEGFR-2 expression *via* IL-6

**Figure 6:**
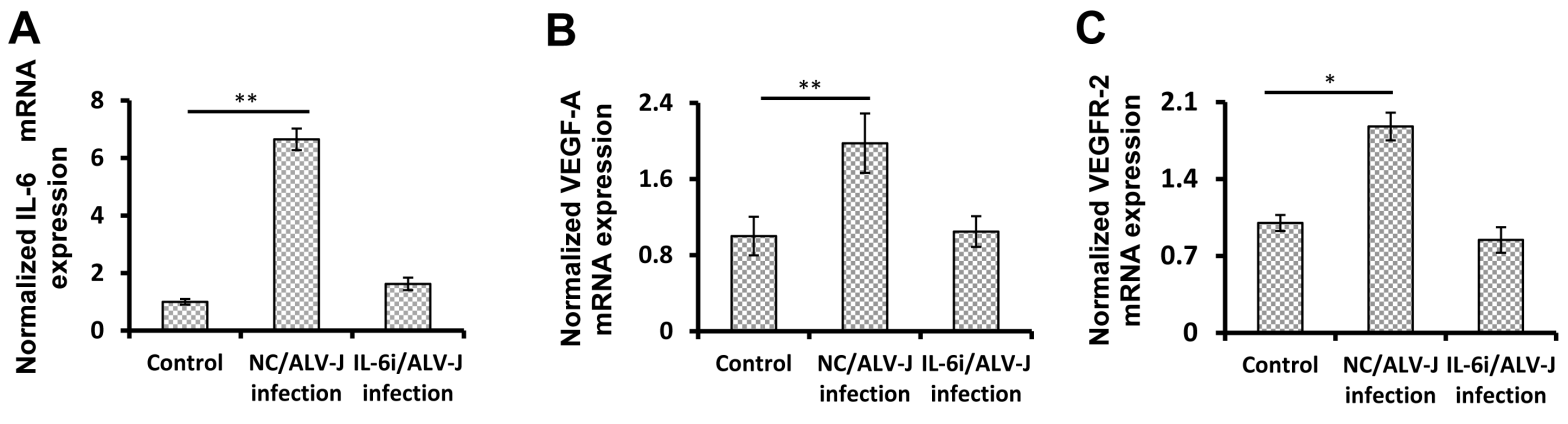
ALV-J increases VEGF-A and VEGFR-2 expression by inducing IL-6 expression **A.** VECs were transfected with *IL6* siRNA or negative control siRNA for 24 h, and then the transfected VECs were infected with or without ALV-J (MOI = 0.1) for another 48 h. Total RNAs were extracted from the VECs and the mRNA levels of *IL6* (to test the effect of the *IL6* siRNA) **A.**, VEGF-A **B.**, and VEGFR-2 were determined by real-time RT-PCR.

The above results have indicated that ALV-J induces IL-6 expression, which stimulates VEGF-A and VEGFR-2 expression. Since we have previously shown that ALV-J increases VEGF-A and VEGFR-2 expression [14], we next explored whether IL-6 represented the key factor inducing the VEGF-A/VEGFR-2 expression in response to ALV-J infection. VECs transfected with IL-6 siRNA or negative control were infected with ALV-J. IL-6 siRNA-transfected VECs had significantly decreased IL-6 mRNA compared with cells transfected with control siRNA (Figure 6A). Infection with ALV-J increased the VEGF-A expression by approximately 2-fold (*p* < 0.01) in cells transfected with control siRNA. However, in cells transfected with IL-6 siRNA, ALV-J failed to induce VEGF-A expression (Figure 6B). Quantitative VEGFR-2 mRNA determination showed a similar result (Figure 6C). Together, these results demonstrate that ALV-J induces VEGF-A and VEGFR-2 expression *via* IL-6 in VECs.

## DISCUSSION

Previous studies have demonstrated that IL-6 induces *in vivo* tumor growth in prostate, breast, and lung cancers [35], and that elevated IL-6 levels stimulate development of inflammation-associated cancers [36, 37]. As we have found that ALV-J infection promotes IL-6 expression *in vivo* [28], we hypothesized that IL-6 might be associated with ALV-J pathogenesis. To test this hypothesis, in the present study, we have examined the ability of ALV-J to induce the IL-6 expression *in vitro*, and we have analyzed the biological impact of the induced IL-6. Our results demonstrate that ALV-J induces VEGF-A and VEGFR-2 expression through activating the IL-6 expression. As VEGF-A and VEGFR-2 are important pro-tumorigenic factors, our results suggest that IL-6 induces ALV-J tumorigenesis through its induction of VEGF-A and VEGFR-2 expression.

We initially demonstrated that ALV-J increased IL-6 expression in vitro. In both splenocytes and PBLs, the induction of IL-6 expression in ALV-J infected cells was brief and transient. However, in VECs, the ALV-J-induced IL-6 expression was persistent and remained increased over time. As splenocytes and PBLs are immune cells, we suspect that there might exist additional immune responses in these cells that regulate the IL-6 expression. In VECs, which do not exhibit pleiotropic immune functions, however, the ALV-J-induced IL-6 increase was continuous. Although the effects of ALV-J appeared to be effectively countered by host immune responses in splenocytes and PBLs, over the course of long-term ALV-J infection *in vivo*, ALV-J may overcome host immune responses to develop a chronic infection. Such a process might be conditional; e.g., affected by the host immune conditions; which is evinced by the low oncogenicity of ALV-J. However, this assumption requires further investigation.

Envelope and capsid proteins are important proteins for ALV-J infection. Gp85 protein is responsible for recognizing specific virus receptors on host cell membranes, and determines the specificity of different subgroups and host range [38-40]. Notably, it has previously been reported that the HIV envelope protein gp120 increases the production of IL-6 in host immune cells, thereby suppressing the immunity of the host [41]. Similarly, in the current study, we found that the ALV-J envelope protein gp85 also induced the production of IL-6 in chicken splenocytes. In addition, we demonstrated that ALV-J capsid protein p27 could be recognized by splenocytes and stimulate IL-6 expression as a host immune response to ALV-J infection. Capsid protein also plays a major role in virus infections; for example, it has been shown to trigger host innate immune responses to HIV *via* interacting with tripartite motif protein isoform 5 alpha [42, 43]. In comparison, for ALV, p27 acts as a major group-specific antigen [44] and an indicator of ALV infection [45-48]. A prior study has suggested that ALV p27 is involved in the immunosuppression induced by ALV in chickens [49].

This study also demonstrated that PI3K and NF-κB signaling pathways were involved in the ALV-J-induced IL-6 expression. NF-κB plays a central role in the activation of numerous pro-inflammatory cytokines in multiple cell types, including macrophages, T cells, and epithelial cells [50]. Similarly to our study, Ankit *et al.* reported that the HIV-1 envelope protein gp120 induced IL-6 expression through an NF-κB-dependent mechanism [41]. Other studies have also indicated that IL-6 expression is regulated *via* NF-κB [51-53]. Consistent with our results, chicken IL-6 promoter sequence (GenBank: AJ250838.2) contains a putative NF-κB binding site (using TRANSFAC). Thus, we speculate that IL-6 is a target gene for NF-κB. In addition, the PI3K signaling pathway is closely related to tumorigenesis *via* inhibiting apoptosis [54] and promoting angiogenesis [55]. In this study, inhibition of PI3K or NF-κB signaling suppressed the ALV-J-induced IL-6 production, suggesting that both pathways are essential for IL-6 expression, and that a cross talk might exist between these two pathways.

When investigating the significance of ALV-J-induced IL-6 expression, we first demonstrated that IL-6 transfection was able to promote VEGF-A and VEGFR-2 expression in VECs. IL-6 promotes VEGF-A and VEGFR-2 production in humans *via* trans-signaling, where soluble and extracellular IL-6 initially binds the surrounding soluble IL-6 receptors and then activates gp130 protein on the surface of target cells to trigger a series of responses [56]. Based on this theory, the embryo assays performed in this study further indicated that the IL-6 present in blood was able to increase VEGF-A and VEGFR-2 expression *in vivo*. Furthermore, our results indicated that STAT3 was involved in this process. Many studies have indicated that VEGF-A and VEGFR-2 are important for tumor growth in different cancers [57-61]. Previous studies in our laboratory have shown that some ALV-J strains have stronger oncogenicity in hemangiomas than other ALV-J strains, with their higher VEGF-A and VEGFR-2 expression in vascular tissues [62]. However, further studies are required to validate the correlation between VEGF and ALV-J oncogenesis.

In summary, the present study indicates that ALV-J increases IL-6 expression, which then induces VEGF-A and VEGFR-2 production. These results suggest that IL-6 plays a central role in ALV-J-induced VEGF-A and VEGFR-2 expression. IL-6 is a proinflammatory cytokine with important roles in inflammation as well as in innate immunity. Innate immune response provides the first line of defense against invading pathogens, leading to the halt of initial spread of infection and activating adaptive immunity and other secondary defense mechanisms [63, 64]. However, many viruses have evolved to hijack the host immune system to facilitate their own proliferation and invasion, such as human herpesviruses, hepatitis C virus, Epstein Barr virus, and human papillomavirus [26, 27]. Inflammation and immunity have been suggested to affect different stages of cancer development, with inflammation and innate immunity exerting protumorigenic effects [65]. Based on our results, we speculate that ALV-J hijacks IL-6 as a means of its tumorigenicity. These findings provide new insight into the tumorigenic mechanisms of ALV-J and suggest potential targets for the development of prophylactic or treatment strategies to mitigate ALV-J infection and pathogenicity.

## MATERIALS AND METHODS

### Cells, viruses, and virus preparation

Splenocytes were isolated from the spleens of 21-day-old specific-pathogen-free (SPF) chickens as described previously [66], and maintained in RPMI 1640 supplemented with 10% FBS, penicillin-streptomycin, and pyruvic acid sodium salt. PBLs were isolated from the anticoagulate of 21-day-old SPF chickens and maintained in RPMI 1640 supplemented with 10% FBS, penicillin-streptomycin, and pyruvic acid sodium salt. VECs were isolated from the 14-day-old SPF embryos as described previously [62], and maintained in Dulbecco's minimum essential medium (DMEM) supplemented with 20% FBS and penicillin-streptomycin.

ALV-J strain HLJ09SH02 was isolated and stored at −70°C in our laboratory and propagated in DF-1 cells as described [67].

### Construction and expression of ALV-J structural protein gene expression vectors and IL-6 gene expression vector

The ALV-J genes for structural proteins including p27, gp85, gp37, reverse transcriptase, and integrase of ALV-J strain HLJ13SH01 were amplified by PCR using specific primers and the PCR products were subcloned into the pcAGGS vector (Addgene) with a Flag tag at the C-terminus. All construction vectors were confirmed by sequencing as compared with ALV-J strain HLJ13SH01 sequence, GenBank accession number KM376510.1), and the protein expression of each was confirmed by western blot (Figure 2A). The IL-6 gene was amplified from chicken spleen using specific primers and the PCR product was then subcloned into the pcAGGS vector. All primers used in this study are listed in Table 1. IL-6 expression was confirmed by ELISA (data not shown).

**Table 1:** Sequences of the primers and probes used in the study

Name	Sequences
p27 forward	5’-GCCGGTACCATGCCTGTAGTGATTAAGACAGAGG-3’
p27 reverse	5’-CAACTCGAGTCAGGCCGCGGCTATGCCTCG-3’
gp85 forward	5’-GCCATCGATATGGGAGTTCATCTGTTGCA-3’
gp85 reverse	5’-CAACTCGAGTCAGCGCCTGCTACGGCGGT-3’
gp37 forward	5’-GAAGGTACCATGTCGCTGAGTCGTCTCTCGCC-3’
gp37 reverse	5’-GCCCTCGAGTCACAGTTGCTCCCTAATTCTAT-3’
IN forward	5’-GCCATCGATATGCCCTTGAGAGAGGCTAAA-3’
IN reverse	5’-GAACTCGAGTCATGCAAAGAGAGGACTCGC-3’
RT forward	5’-GCCGGTACCATGACTGCTGCGCTACATCTG-3’
RT reverse	5’-GGCCTCGAGTCAATACGCTTGAAAGGTGGC-3’
IL-6 forward	5’-GCCGGTACCGTGTGCAGCGGTTGAACC-3’
IL-6 reverse	5’-CGGCTCGAGAGTTCATGGTGCGGCTTC-3’

Abbreviations: IN: integrase; RT: reverse transcriptase

### IL-6 mRNA quantification

Splenocytes, PBLs, and VECs were infected with ALV-J strain HLJ09SH02 at a multiplicity of infection (MOI) of 0.1. To confirm successful ALV-J infection, cells infected with ALV-J were collected at 3, 6, 12 and 24h post-infection. Cell lysates were prepared after three freeze-thaw cycles and tested for ALV group specific antigen by an antigen capture enzyme-linked immunosorbent assay with anti-p27 antibody-coated plates (IDEXX Inc., MA).

Total RNA was extracted from the infected cells using TRIzol reagent (Invitrogen Life Technologies, Carlsbad, CA, USA) and then purified using the RNeasy Mini kit (Qiagen, Venlo, The Netherlands). Real-time RT-PCR was performed on the Roche LightCycler real-time PCR system (Madison, WI, USA) using specific primers and probe for chicken IL-6 (forward 5′-GAT GGG ACG GCG GCC GGG GAG-3′, reverse 5′- TAA CGG CGG CGG GCA GCG GGA-3′, probe 5′-(FAM)-GGA CGG GGC GCT CTC CGG CGG-(TAMRA)-3′) and the One Step PrimeScript RT-PCR Kit (Perfect Real Time) (TaKaRa, Dalian, China) following the manufacturer's instructions. IL-6 gene expression was normalized to that of the 28S rRNA gene (forward 5′-ATC CTG CCA GTA GCA TAT G-3′, reverse 5′-GCC GTG CGT ACT TAC ACG T-3′, probe 5′-(FAM)-GCA TGG CTT AAT CTT TGA GAC AA-(TAMRA)-3′) and presented as fold induction relative to medium control.

Splenocytes seeded in 12-well plates were transfected with p27, gp85, gp37, reverse transcriptase, and integrase gene expression vectors (2 μg/5 × 10^6^ cells) using Lonza Nucleofector Kits. IL-6 mRNA was measured at 48 h post transfection as described above.

To determine whether ALV-J induced VEGF-A and VEGFR-2 expression *via* IL-6, VECs were transfected with IL-6 siRNA or negative control siRNA and 24 h later were infected with ALV-J over another 24 h. The expression of IL-6 was then tested as described above to confirm siRNA efficacy.

### ELISA for IL-6

The levels of secreted IL-6 were measured in cell culture supernatants using commercially available chicken IL-6 ELISA kits (Uscn Life Science Inc., Aachen, Germany) according to the manufacturer's instructions.

### Inhibition of signal transduction pathways

Splenocytes were pretreated with control DMSO, PI3K inhibitor LY294002 (Enzo Life Sciences; 5 μM), MEK inhibitor PD98059 (Enzo Life Sciences; 10 μM), p38 mitogen-activated protein kinase (MAPK) inhibitor SB203580 (Enzo Life Sciences; 5 μM), PKC inhibitor GF-109203X (Enzo Life Sciences; 1 μM), or NF-κB inhibitor BAY11-7082 (Enzo Life Sciences; 1 μM) for 1 hour as described (68), and then infected with ALV-J at an MOI of 0.1. After 48 h, supernatants were collected for IL-6 assay by ELISA, and cells were harvested for IL-6 mRNA analysis by real-time RT-PCR.

### Dual-Glo luciferase assay

Splenocytes seeded on a 12-well plate were pretreated with the PI3K inhibitor LY294002 (5 μM) or equivalent DMSO for 1 h. Then the cells were transfected using Lonza Nucleofector Kits with NF-κB activity test plasmids (including a luciferase signal, provided by professor Xiaojun Wang) together with pRL-TK control plasmid (Promega) and simultaneously infected with ALV-J (MOI = 0.1) or DMEM. After another 6 h, the cells were harvested for luciferase analysis with Dual-Glo Luciferase Assay System (Promega) following manufacturer's instructions.

### VEGF-A and VEGFR-2 mRNA quantification

To test the effect of IL-6 on VEGF-A and VEGFR-2 expression, VECs pretreated with the STAT3 inhibitor stattic (Santa Cruz Biotechnology; 5 μM) or equal volume of DMSO were transfected with the IL-6 gene expression vector for 48 h. Total RNA was extracted using TRIzol and purified using RNeasy Mini kit. Real-time RT-PCR was performed using specific primers for chicken VEGF-A (forward 5′-GTC GTA CAT ATT CAG GCC ATC-3′, reverse 5′-GAT TCT TTG GTC TGC AGT CAC-3′) or VEGFR-2 (forward 5′-TAA GGC ATC CAA CCA GAC AAG-3′, reverse 5′-GTA CTA GAG TGG CGG GGA CAC-3′) and the One Step SYBR PrimeScript RT-PCR Kit II (Perfect Real Time) (TaKaRa) following the manufacturer's instructions using the Roche LightCycler real-time PCR system. VEGF-A and VEGFR-2 gene expression was normalized to 28S rRNA and presented as fold induction relative to medium control.

Embryos inoculated with recombinant IL-6 protein (1 μg/embryo) or PBS at equal volumes were used to test the effect of IL-6 on VEGF-A and VEGFR-2 expression *in vivo*. At 1, 2, and 3 days after inoculation, three embryos were tested each from the control and infected groups. RNAs were isolated from the vascular tissues of the embryos and used to test mRNA expression by real-time RT-PCR as described above.

To explore whether ALV-J induced VEGF-A and VEGFR-2 expression *via* IL-6, VECs were transfected with IL-6 or negative control siRNA and infected with ALV-J 24 h later for an additional 24 h. The expression of VEGF-A and VEGFR-2 were then tested as described above.

### SOCS3 mRNA quantification

To further study whether the STAT3 pathway was activated upon IL-6-induced VEGF-A and VEGFR-2 expression, VECs were transfected with the IL-6 gene expression vector or control vector for 48 h. Total RNA was extracted and purified as previously described. Real-time RT-PCR was performed using specific primers for chicken SOCS3 (forward 5′-TCA GCT CTA AGA GCG AGT ACC-3′, reverse 5′-GCT GAG GGT GAA GAA GTG C-3′) or VEGFR-2 (forward 5′-TAA GGC ATC CAA CCA GAC AAG-3′, reverse 5′-GTA CTA GAG TGG CGG GGA CAC-3′) and the One Step SYBR PrimeScript RT-PCR Kit II (Perfect Real Time) according to manufacturer instruction for the Roche LightCycler real-time PCR system. SOCS3 gene expression was normalized to 28S rRNA and presented as fold induction relative to medium control.

Embryos inoculated with recombinant IL-6 protein (1 μg/embryo) or equivalent volumes of PBS were also used to test the activation of the STAT3 signaling pathway. On 1, 2, and 3 days after inoculation, three embryos were tested each from the control and infected groups. RNAs were isolated from the vascular tissues of the embryos and were used to test the expression of SOCS3 mRNA by real-time RT-PCR as described above.

### Statistical analysis

All experiments were performed with at least three independent replicates and the data are reported as the means ± standard deviation (SD). Differences in data were evaluated by the Student's t test. A *p* value of < 0.05 was considered to be statistically significant.
